# Radiographic imaging in pediatric suspected foreign body ingestion: potential for dose reduction through collimation and exposure strategies

**DOI:** 10.1007/s00247-026-06751-7

**Published:** 2026-08-20

**Authors:** Max-Johann Sturm, Clemens Benoit, Jan-Peter Grunz, Stefan Weick, Franziska Müller-Reichart, Anke Dick, Oliver Andres, Thorsten Alexander Bley, Henner Huflage

**Affiliations:** 1 Department of Diagnostic and Interventional Radiology, University Hospital Würzburg, Oberdürrbacher Str. 6, Wurzburg, D-97080 Germany; 2 Department of Radiation Oncology, University Hospital Würzburg, Wurzburg, Germany; 3 Department of Pediatrics, University Hospital Würzburg, Wurzburg, Germany

**Keywords:** Child, Foreign bodies, Gastrointestinal tract, Magnets, Radiography, Radiation protection

## Abstract

**Background:**

Foreign body ingestion constitutes a common emergency in children. Radiography is essential for confirming presence, type, and location of ingested objects. International guidelines recommend broad anatomical coverage from the neck to the pelvis; however, the impact of radiographic exposure parameters and collimation on radiation dose remains underexplored.

**Objective:**

To evaluate the effects of different scan protocols on radiation dose in pediatric foreign body ingestion.

**Materials and methods:**

Two-hundred-twenty-one radiographs from 183 pediatric patients (median age 2.8 years [interquartile range 1.5–5.5 years]; 112 males, 71 females) performed between September 2019 and April 2025 were retrospectively analyzed. Radiographs were categorized as neck–upper abdomen, extended collimation including pelvis, or abdomen only. Notably, the first two protocols used chest radiograph exposure parameters. Data included patient demographics, foreign body type and location, radiographic protocol, dose-area product, and estimated effective dose.

**Results:**

A foreign body was detected in 55.7% of radiographs, most of them in the stomach (59.4%). Coins were most common (31.7%), followed by button batteries (13.8%) and magnets (12.2%). Dose-area products were comparable between standard neck-to-upper-abdomen and extended neck-to-pelvis collimation but were markedly higher for abdomen-only imaging (*P*<0.001). Effective dose increased with extended collimation (*P*<0.05) and was highest for abdomen-only radiographs (0.053 mSv [0.034–0.086 mSv], *P*<0.001). No differences were observed in patient age or repeat imaging rates.

**Conclusion:**

In suspected pediatric foreign body ingestion, collimation and exposure parameters substantially influence radiation dose. Low-dose chest parameters combined with restrictive collimation minimize radiation exposure, whereas abdominal radiographs carry higher dose, attributable predominantly to the abdominal exposure parameters employed.

**Graphical abstract:**

**Figure:**
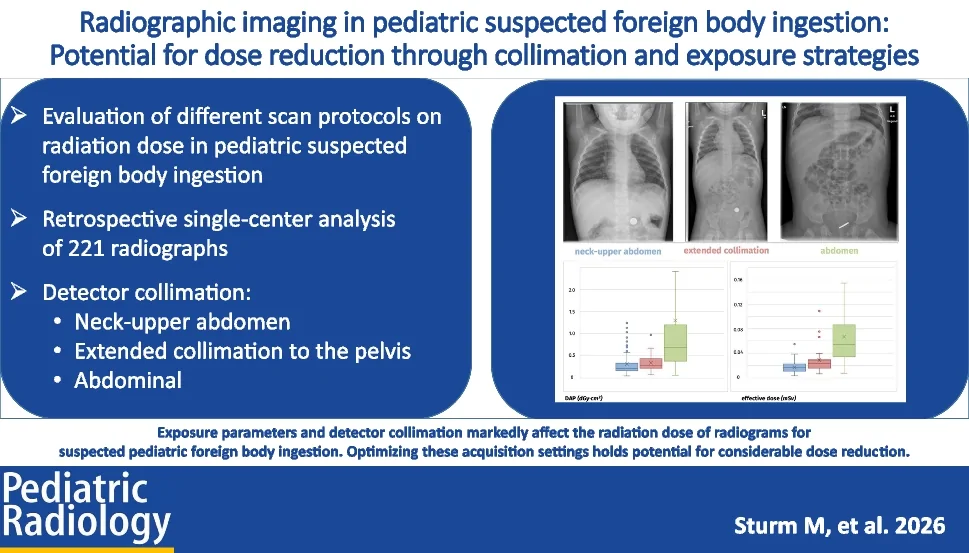

## Introduction

Foreign body ingestion represents a common pediatric emergency, with a reported rising incidence in recent years [1–5]. Foreign body ingestions account for up to 1% of pediatric emergency department visits [3]. In 2016, it was among the five most frequent reasons for U.S. Poison Control Center consultations, with 67,771 cases in children ≤5 years of age [6]. The majority of cases occur in infants and young children [7], which can be attributed, among other factors, to their mouthing behavior and increased object-to-mouth frequency [8, 9]. Pediatric foreign body ingestions are challenging to manage, as they represent a spectrum ranging from harmless objects that often pass spontaneously to potentially life-threatening emergencies [1, 7, 10–13]. Since many foreign bodies, including button batteries, magnets, and coins are radiopaque, radiographic imaging is the diagnostic standard to verify their presence, type, and location [3, 14–16]. Radiography is also essential for identifying and discriminating potentially dangerous situations [17, 18].

A comprehensive radiographic examination of the entire gastrointestinal tract is often recommended in clinical guidelines [11, 13, 17, 19–21], although this recommendation is largely based on expert consensus and clinical practice rather than empirical evidence. Conversely, the “as low as reasonably achievable” (ALARA) principle emphasizes that radiation exposure should be minimized to reduce potential risks [22–25]. Therefore, imaging protocols should be carefully evaluated to balance exposure settings and radiation dose against clinical relevance and potential consequences [26, 27]. A key strategy for dose optimization in pediatric radiography is the use of strict X‑ray beam collimation, which minimizes unnecessary irradiation of non or less relevant anatomical regions [27–29]. In the radiographic management of foreign body ingestion, the need for adequate anatomical coverage should be balanced with the principle of strict beam collimation under ALARA. In certain clinical situations, broader collimation may be justified. Accordingly, we aimed to analyze radiographic practice in suspected pediatric foreign body ingestion, focusing on the dose implications of exposure strategies, including extended beam collimation.

## Methods

### Patient population

Between September 2019 and April 2025, all patients younger than 18 years who underwent radiographic imaging for suspected foreign body ingestion at the department of pediatric radiology of a tertiary university hospital were retrospectively identified from the hospital information system (SAP, Walldorf, Germany). From an initial 228 radiographs, 7 were excluded due to non-standard projections or incomplete protocols (e.g., neck-only collimation or lateral projection), leaving 221 anteroposterior radiographs for analysis. Consecutive patients with anteroposterior (AP) radiography were included. Repeat radiographs were included regardless of the time interval to the initial examination, and patients could therefore contribute multiple examinations to the dataset. This occurred for two distinct reasons: repeat imaging within the same clinical episode, or separate emergency department attendances for distinct episodes of suspected foreign body ingestion. Repeat radiographs performed within 6 h of the initial examination were recorded as a surrogate marker of diagnostic adequacy, reflecting potential clinical concern during the active emergency department visit. Repeat radiographs performed beyond 6 h represented separate emergency department attendances, most commonly in cases where spontaneous passage of the foreign body had not been confirmed and were treated as independent examinations. All included radiographs were acquired using a digital radiography system (Ysio Max, Siemens Healthineers, Forchheim, Germany). Radiographs for suspected foreign body ingestion were acquired according to institutional standards in the supine position on a radiographic Bucky table, using an AP projection with a focus-to-detector distance of 115 cm. All examinations were conducted using a 0.2-mm copper prefiltration. The weight-dependent exposure parameters are listed in Table 1 (chest view protocols: 70–125 kV and 0.8–1.0 mAs) and Table 2 (abdominal protocol: 70–85 kV and 3.6–5.0 mAs). Automatic exposure control was applied for children exceeding 30 kg body weight when using chest exposure parameters, and for children exceeding 40 kg body weight when using abdominal exposure parameters.

**Table 1:** Exposure parameters for anteroposterior chest view protocols

Weight	Kilovolt	mAs	Anti-scatter-grid
<3 kg	70	0.8	No
3–7 kg	73	0.8	No
7–15 kg	75	1.0	No
15–30 kg	81	1.0	No
30–40 kg	102	1.0	Yes
40–50 kg	109	1.0	Yes
50–70 kg	113	1.0	Yes
>70 kg	125	1.0	Yes

Both neck–upper abdomen and extended collimation from the neck to the pelvis examinations were performed with an additional copper prefiltration of 0.2 mm and a film-focus-distance of 115 cm

**Table 2:** Exposure parameters for abdomen protocol

Weight	Kilovolt	mAs	Anti-scatter-grid
<3 kg	70	3.6	No
3–7 kg	70	4.5	No
7–15 kg	73	4.5	No
15–25 kg	75	5.0	No
25–40 kg	77	5.0	No
40–50 kg	81	5.0	Yes
50–70 kg	85	5.0	Yes
>70 kg	85	5.0	Yes

Radiographic exposure parameters for abdomen supine. All images were acquired with an additional copper prefiltration of 0.2 mm and a film-focus-distance of 115 cm

### Collimation and radiation dose

**Fig. 1:**
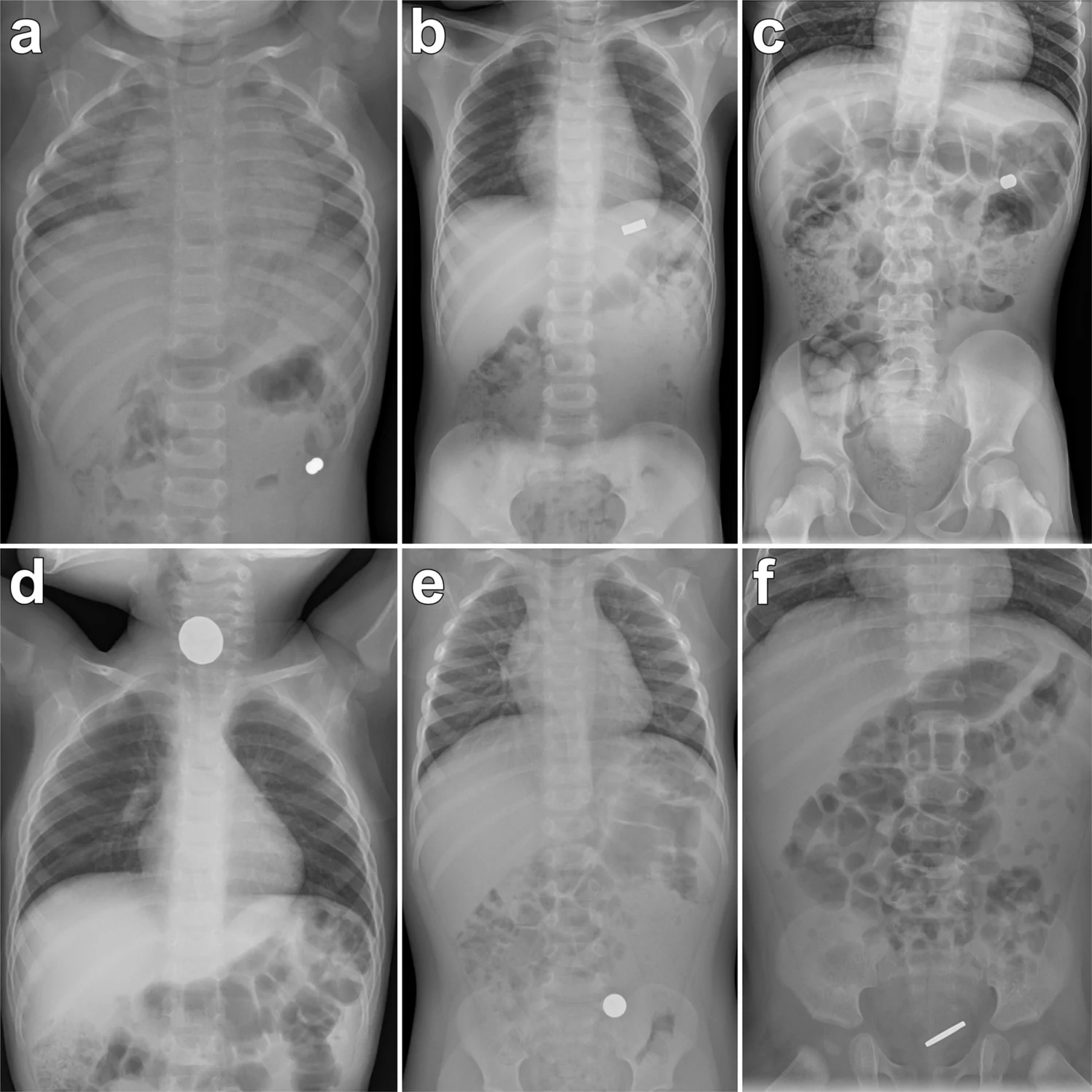
Ingested foreign bodies — magnets (**a**–**c**) and coins (**d–f**) — detected on anteroposterior radiographs in six different children. **a**, **d** Neck–upper abdomen protocol in two 1-year-old boys. **b**, **e** Extended collimation protocol in a 5-year-old boy (**b**) and a 1-year-old boy (**e**). **c**, **f** Abdomen protocol in a 6-year-old boy (**c**) and a 1-year-old boy (**f**)

**Fig. 2:**
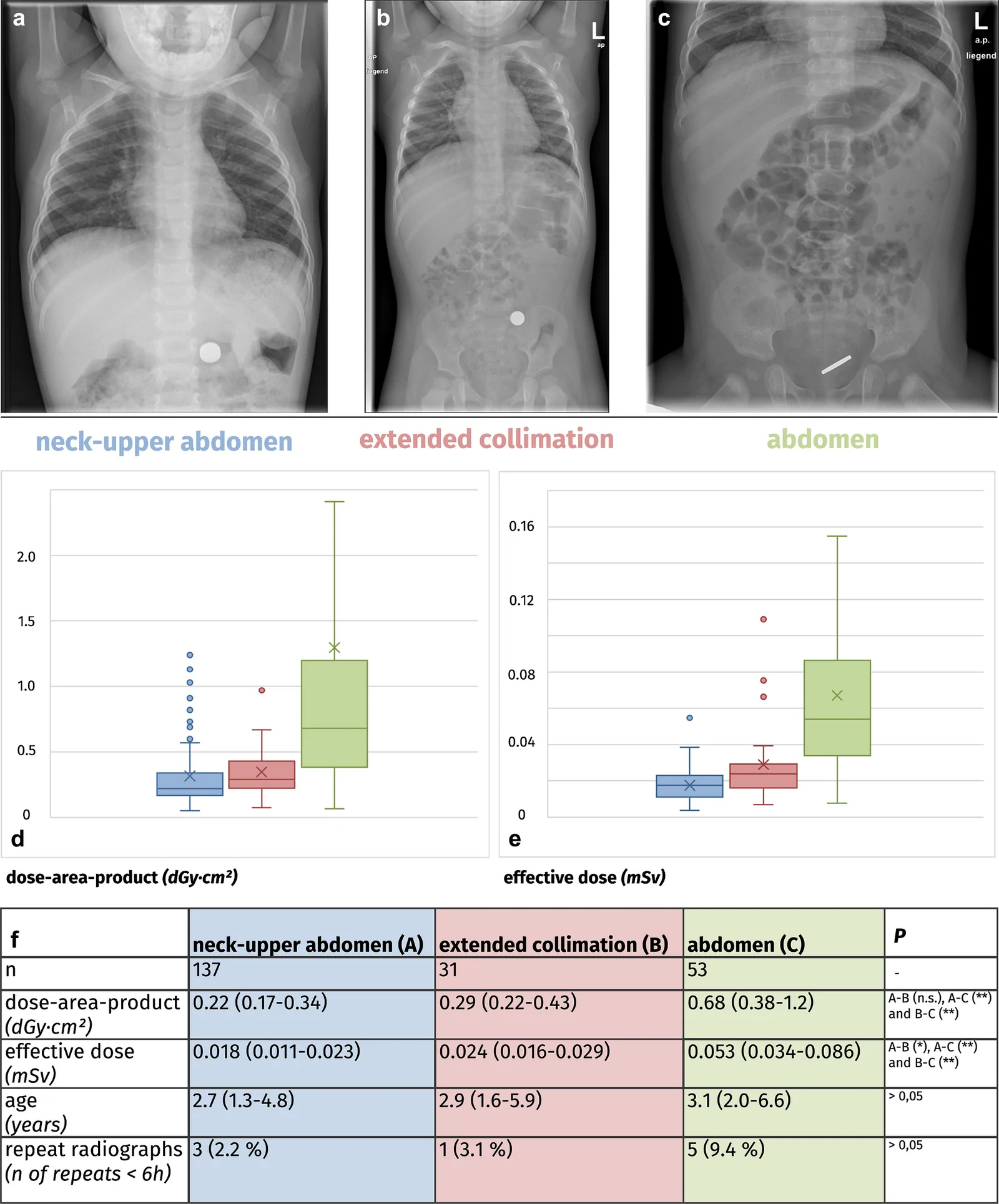
Overview of protocol groups and associated radiation doses. Anteroposterior radiographs of ingested foreign bodies acquired with three different collimation protocols: **a** neck-upper abdomen protocol in a 1-year-old girl with an ingested button battery. **b** Extended collimation protocol in a 1-year-old boy with an ingested coin. **c** Abdomen protocol in a 1-year-old boy with an ingested coin. Box plots of dose-area product (**d**) and effective dose (**e**) per protocol group, presented as median with interquartile range (*n*=137, 31, and 53 for groups A, B, and C, respectively). **f** Summary of protocol group characteristics and associated radiation doses, presented as median with interquartile range; *P*-values refer to pairwise comparisons following the Kruskal-Wallis test with Bonferroni correction

Collimation was classified into three categories: (A) neck to upper abdomen; (B) extended collimation from the neck to the pelvis, with visible iliac crests as the minimum criterion; and (C) abdomen only (Figs. 1 and 2). All included radiographs, regardless of group, were exclusively acquired for the indication of suspected foreign body ingestion. The dose–area product (DAP) of each radiographic examination was retrieved from the automated dose reports or the respective scan’s DICOM metadata. The effective dose of each radiographic examination was estimated by multiplying the DAP by an age-dependent conversion factor specific to the respective anatomical region (expressed in mSv/[dGy·cm^2^]). When examinations encompassed multiple anatomical regions (e.g., neck, chest, abdomen, or abdomen and pelvis), the arithmetic mean of the corresponding regional and age-dependent conversion factors was used to derive a single conversion factor. This approach is based on the recommendations of the International Commission on Radiological Protection, particularly ICRP Publication 103 (2007), which serves as a standard reference for effective dose estimation in current literature [30–35]. Conversion factors were derived from the institutional dose management system (DOSE, Quaelum NV, Leuven, Belgium), which provides region-specific factors in accordance with ICRP 103. For predefined anatomical combinations available within the system, the combined conversion factor corresponds to the arithmetic mean of the constituent regional factors, thereby internally validating this averaging approach. For collimation regions not available as predefined combinations, the arithmetic mean of the respective regional factors was calculated accordingly. The age-dependent conversion factors are listed in Table 3 and Table 4.

**Table 3:** Age-dependent conversion factors

Age (years)	Neck	Chest	Abdomen	Pelvis
0–1	0.089	0.307	0.248	0.248
1–3	0.0439	0.176	0.115	0.115
4–7	0.0254	0.061	0.072	0.072
8–12	0.0271	0.0469	0.0453	0.0453
13–17	0.0095	0.0265	0.0248	0.0248
>18	0.0103	0.0212	0.0202	0.0202

Conversion factors are based on the International Commission on Radiological Protection (ICRP) Publication 103 (2007) in mSv/dGy*cm^2^ (ICRP 103 also provides combined conversion factors for multiple regions, those resemble the arithmetic mean)

*ICRP*, International Commission on Radiological Protection

**Table 4:** Age-dependent conversion factors used for the estimation of the effective dose of the three collimation groups in our study based on the arithmetic mean of conversion factors provided by the ICRP Publication 103 (2007) in mSv/dGy*cm^2^

Age (years)	Group A	Group B	Group C
0–1	0.198	0.223	0.248
1–3	0.10995	0.112475	0.115
4–7	0.0432	0.0576	0.072
8–12	0.037	0.04115	0.0453
13–17	0.018	0.0214	0.0248
>18	0.01575	0.017975	0.0202

*Group A* neck to upper abdomen (neck, chest), *Group B* extended collimation to the pelvis (neck, chest, abdomen, pelvis), *Group C* abdominal radiographs (abdomen, pelvis) (The conversion factor for each anatomical region was included only if the majority of that region was within the collimation field. In Group A, since most of the abdomen was outside the collimation field, only the conversion factors for the neck and chest regions were included in the calculation.)

*ICRP* International Commission on Radiological Protection

### Foreign bodies

Retrospective analyses of foreign bodies, including type and anatomical location, as well as collimation and radiation dose, were based on data extracted from the radiographs, corresponding radiology reports, and clinical information stored in the Picture Archiving and Communication System (PACS; MERLIN, Phönix PACS GmbH, Freiburg im Breisgau, Germany). Foreign bodies, when present, were classified as coins, button batteries, magnets, or other foreign bodies. The anatomical location was categorized as cervical, thoracic, gastric, or post-pyloric based on radiographic projection.

### Statistical analysis

Statistical analyses were performed using SPSS version 31.0 (IBM, Armonk, NY, USA). The collimation groups were tested for normal distribution using the Shapiro–Wilk test. As data were not normally distributed, continuous variables are reported as median values and interquartile ranges. Differences between groups were assessed using the Kruskal–Wallis test for independent samples. Following significant Kruskal-Wallis tests, pairwise comparisons were performed using Dunn’s post-hoc test with Bonferroni correction for multiple testing. Repeat examination rates were compared using the Fisher exact test. Pairwise effect sizes for significant Kruskal-Wallis comparisons were quantified using Cliff’s delta (*δ*) with corresponding 95% confidence intervals calculated in R (version 4.5.2; R Foundation for Statistical Computing, Vienna, Austria). The primary outcome measure was the effective dose associated with different collimation strategies. For all tests, *P*-values below 0.05 were assumed to indicate statistical significance.

## Results

### Patient sample and foreign bodies

**Fig. 3:**
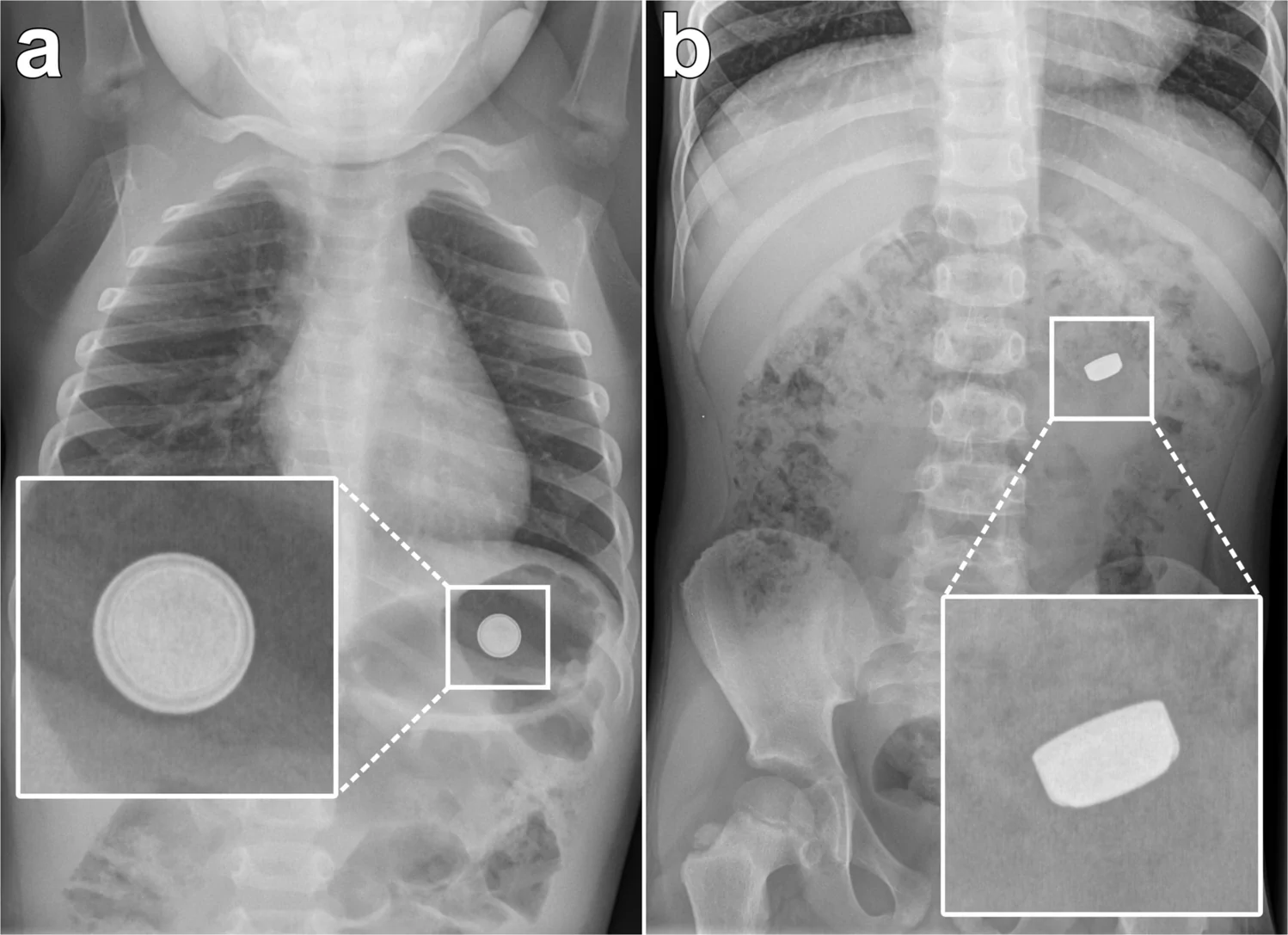
Ingested button batteries (**a**,** b**) detected on anteroposterior radiographs in two children. The characteristic double-rim sign is clearly visible in both images. **a** Neck–upper abdomen protocol in a 1-year-old girl. **b** Abdomen protocol in a 6-year-old boy

A total of 221 radiographs from 183 individual patients were included in the analysis. Of these, 112 patients were male (61.2%) and 71 were female (38.8%). The median age was 2.8 years (interquartile range 1.5–5.5 years). A radiopaque foreign body was detectable in 123 radiographs (55.7%). Ingested coins were the most common foreign bodies (*n*=39, 31.7%), followed by button batteries (*n*=17, 13.8%) and magnets (*n*=15, 12.2%). All button batteries detected during the study period were clearly identifiable on AP projections based on their characteristic shape and/or the presence of a double-rim sign (Fig. 3). Other foreign bodies (*n*=52, 42.3%) comprised, for example, jewelry or necklaces, screws, or beverage can caps. The most common anatomic location of a foreign body was gastric (*n*=73, 59.4%), followed by post-pyloric (*n*=41, 33.3%). Eight foreign bodies (6.5%) were located at the upper esophageal sphincter, and one foreign body (0.8%) was seen in the thoracic projection. Foreign body data are summarized in Table 5 and exemplary images are given in Fig. 1.

**Table 5:** Patient sample and foreign bodies

**Radiographs**	221
**Patients**	183
Male	112 (61.2%)
Female	71 (38.8%)
**Age** (median, interquartile range)	2.8 years, 1.5–5.5 years
**Detected foreign bodies**	123/221 (55.7%)
**Kind of foreign body**	
Coin	39 (31.7%)
Button battery	17 (13.8%)
Magnet	15 (12.2%)
Other	52 (42.3%)
**Anatomic location**	
Neck/upper esophageal sphincter	8 (6.5%)
Thoracic	1 (0.8%)
Gastric	73 (59.4%)
Postpyloric	41 (33.3%)

### Radiographic collimation and radiation dose

Group A radiographs (i.e., neck to upper abdomen) were most common, comprising 137 radiographs (median patient age of 2.7 years; interquartile range 1.3–4.8 years). DAP and effective dose were 0.22 dGy·cm^2^ (0.17–0.34 dGy·cm^2^) and 0.018 mSv (0.011–0.023 mSv), respectively. Group B, characterized by extended collimation to the pelvis with constant radiographic exposure parameters, included 31 radiographs with a median patient age of 2.9 years (1.6–5.9 years). The DAP in this group was 0.29 dGy·cm^2^ (0.22–0.43 dGy·cm^2^), corresponding to an effective dose of 0.024 mSv (0.016–0.029 mSv). In group C, 53 radiographs with a median patient age of 3.1 years (2.0–6.6 years) received abdominal radiographs. Median DAP of these scans was 0.68 dGy·cm^2^ (0.38–1.20 dGy·cm^2^), corresponding to an effective dose of 0.053 mSv (0.034–0.086 mSv).

No significant difference in DAP was observed between groups A and B, both acquired with identical radiographic exposure parameters according to institutional standard (Table 1; tube current–time product 0.8–1 mAs), despite the extended collimation in group B. In contrast, abdominal radiographs in group C, acquired with higher exposure parameters (Table 2; tube current–time product 3.6–5 mAs), showed a marked increase in DAP compared with both other groups (*P*<0.001). Pairwise effect size analysis demonstrated a small effect for DAP between groups A and B (*δ*=−0.247, 95% confidence interval −0.457 to −0.011) and large effects for comparisons involving abdominal radiographs (groups A vs. C: *δ*=−0.680, −0.794 to −0.519; groups B vs. C: *δ*=−0.585, −0.748 to −0.355). Notably, group B with extended collimation showed a higher effective dose than group A (*P*<0.05). Furthermore, the effective dose of abdominal radiographs in group C was substantially higher than effective doses in groups A and B (*P*<0.001). For effective dose, the effect size was moderate between groups A and B (*δ*=−0.371, 95% confidence interval −0.575 to −0.124) and large for comparisons involving abdominal radiographs (groups A vs. C: δ=−0.855, −0.928 to −0.719; groups B vs. C: δ=−0.631, −0.790 to −0.392).

No significant difference in patient age was ascertained among groups (*P*=0.204). Foreign bodies were detected in 76 radiographs in group A (55.5%), 15 radiographs in group B (48.4%), and 32 radiographs in group C (60.4%). Repeated radiographic examinations performed within 6 h of the initial radiograph occurred three times in group A (2.2%), once in group B (3.2%), and five times in group C (9.4%), with no significant differences between groups (*P*=0.078). However, no foreign body was detected on any of the repeat radiographs. An overview of the results is presented in Fig. 2.

## Discussion

Radiography remains the primary imaging modality for suspected foreign body ingestion in children worldwide [13, 19]. Despite broad international recommendations advocating comprehensive anatomical coverage from the neck to the pelvis, there is a notable lack of evidence addressing technical acquisition parameters that may allow radiation dose optimization in this frequent pediatric emergency. In particular, data on how exposure settings and collimation strategies influence radiation dose in routine clinical practice are scarce. Our study demonstrates that both radiographic exposure settings and the extent of collimation substantially influence radiation dose in pediatric patients with suspected foreign body ingestion. Abdominal radiographs acquired with standard abdominal exposure parameters showed higher dose metrics than chest-based radiographs, even when the latter included extended collimation to the pelvis. When compared directly, extending collimation from the neck to the pelvis using identical chest exposure parameters significantly increased the effective dose compared with collimation limited to the neck, chest, and upper abdomen. These results highlight clear opportunities for dose reduction through careful adjustment of exposure parameters and justified collimation. These results highlight clear opportunities for dose reduction through careful adjustment of exposure parameters and justified collimation. Although all three protocols delivered effective doses well below 0.1 mSv, approaching the range of a few days of natural background radiation, dose minimization remains relevant given the radiosensitivity of pediatric patients, their longer life expectancy, and the absence of a proven safe dose threshold under the linear no-threshold model [36, 37]. It should be acknowledged, however, that the linear no-threshold model and its application at very low doses remain subject to ongoing scientific debate, with some data suggesting possible threshold or hormetic effects at the sub-0.1 mSv level [38]. Nevertheless, in the absence of conclusive evidence to the contrary, the ALARA principle remains the appropriate guiding framework for pediatric radiation protection.

Abdominal radiographs exhibited the highest dose metrics among the examined imaging groups. Since both the abdominal protocol and the guideline-based foreign body survey acquired with chest exposure parameters are routinely used in current clinical practice, the exposure differences observed in this study highlight a practical opportunity for dose optimization in routine care. It is well established that abdominal radiographs are associated with higher radiation doses than chest radiographs, as reflected in diagnostic reference levels [24, 39, 40]. This difference is largely attributable to exposure settings designed to provide broadly diagnostic image quality across a wide range of abdominal indications [26]. However, in the context of suspected foreign body ingestion, where the primary diagnostic task is often the detection of high-contrast, radiopaque objects, such general-purpose exposure settings may be more conservative than necessary [15, 41].

From a retrospective perspective, the routine use of isolated abdominal radiographs for foreign body detection should therefore be carefully considered. This approach excludes the neck and esophagus, where foreign bodies may remain clinically silent [42]. And it exposes radiosensitive abdominal organs using exposure parameters that may exceed what is required for the specific diagnostic task [23, 24]. These findings support the potential to refine abdominal technique charts for suspected foreign body ingestion, for example through indication-adapted low-dose abdomen protocols using higher tube voltage with reduced tube current-time product, strict collimation, and weight- or habitus-based exposure presets [26, 27]. Establishing and auditing a dedicated, indication-specific exposure pathway for abdominal radiographs in the foreign body work-up may reduce radiation dose without compromising diagnostic confidence, in guideline-based imaging from the neck to the pelvis as well in follow-up examinations. With further optimization, low-dose photon-counting CT may become a diagnostic alternative in selected complex cases. Reports from ventriculoperitoneal shunt imaging demonstrate that sub-millisievert photon-counting CT can achieve effective doses comparable to or even below a conventional radiographic work-up, a trend that may prove equally relevant in complex foreign body ingestion scenarios (e.g., ingestion of multiple magnets) [43–45].

In our cohort, the most common imaging approach was restricted collimation from the neck to the upper abdomen. This reflects the former German guideline for pediatric foreign body ingestion, which recommended a more restrictive primary approach with extension based on clinical judgment, for example in cases involving magnets, until the end of 2024 [46]. Since then, collimation from the neck to the pelvis has become the standard primary workup, aligning with international guidelines, including the American College of Radiology Appropriateness Criteria, which consider radiographic evaluation of the neck, chest, abdomen, and pelvis appropriate in suspected pediatric foreign body ingestion [13, 19, 47]. These circumstances create an opportunity to reevaluate the restrictive collimation strategy, which may represent an effective approach to dose optimization in defined clinical situations.

Strict collimation is a cornerstone of radiation protection in pediatric radiology and a key element of the ALARA principle [27]. In our cohort, extension of collimation towards the pelvis under identical exposure parameters did not result in a significant increase in DAP. These examinations effectively correspond to chest radiographs extended caudally. In contrast, effective dose, which accounts for region-specific tissue weighting factors and the irradiation of radiosensitive organs such as the gonads, was significantly higher with extended collimation [30]. The effective dose increase with caudal field extension is not exclusively attributable to gonadal irradiation. Under ICRP 103, the gonadal tissue weighting factor was reduced from 0.20 in ICRP 60 to 0.08, placing gonads below red bone marrow, colon, lung, stomach, and breast, each weighted at 0.12 [30]. The effective dose increase from group a to group B therefore reflects contributions from multiple radiosensitive structures. Nevertheless, direct gonadal irradiation remains a particular concern in girls, where the ovaries lie within the primary beam and cannot be shielded without compromising diagnostic image quality. Avoiding direct irradiation of the gonads by keeping them outside the primary beam whenever feasible therefore remains an important component of radiation protection in pediatric radiography, particularly given the declining role of gonadal shielding [23, 48, 49]. This finding underscores the importance of justification, with the irradiated volume carefully tailored to the specific clinical question.

Ingested radiopaque foreign bodies in children constitute a heterogeneous group with respect to potential risk and therapeutic implications [13]. High-risk objects, such as multiple magnets, may lead to severe gastrointestinal complications including perforation and may justify extended collimation to assess the entire gastrointestinal tract [50]. In contrast, coins, which represented the most frequently ingested foreign body in our cohort and in the literature, primarily pose a risk when impacted in the pharynx or esophagus [3, 13]. Once a coin has reached the stomach or passed the pylorus, intervention is rarely required. In this context, primary collimation from the neck to the upper abdomen may be an appropriate imaging strategy for selected patients. This approach is supported by previous studies demonstrating that a chest radiograph including the neck and upper abdomen is adequate for children presenting with coin ingestion [42, 51].

Additional dose reduction may be achieved by limiting the number of acquired projections. In our retrospective cohort, a single AP projection was sufficient in all cases, and no additional lateral projections were required. All ingested button batteries were reliably identified on AP radiographs by their characteristic contour and/or “double rim sign” [15, 16]. These findings support the adequacy of single-plane imaging in the majority of pediatric foreign body ingestion. Nonetheless, when localization is uncertain, acquiring a second projection, as recommended in various guidelines, may be useful [20, 50].

This study has several limitations. Its retrospective, single-center, single-vendor design limits generalizability, as imaging practices were influenced by national guidelines and local standard operating procedures that may not reflect practice at other institutions or with different equipment. Protocol assignment was not randomized but determined by the radiologist on duty based on clinical presentation and institutional convention, introducing an inherent selection bias that prevents the three groups from being considered fully exchangeable. Furthermore, protocol C differs from protocols A and B in both collimation field and exposure parameters simultaneously, meaning that the independent contributions of these two factors to the observed dose differences cannot be disentangled from the present dataset. Effective dose was estimated using conversion factors derived by arithmetic averaging of ICRP tissue weighting factors across anatomical regions; an approach that may not perfectly reflect the true organ dose distribution for each collimation field. Determining the absolute minimum achievable exposure parameters for the specific equipment used would require dedicated phantom studies, which were beyond the scope of this work. Finally, a formal diagnostic accuracy analysis was not possible given the unconfirmed nature of the clinical indication and the absence of a reliable reference standard in routine emergency department practice. Repeat radiography within 6 h was used as a surrogate marker of diagnostic adequacy, which, while pragmatic, cannot fully exclude the possibility of missed foreign bodies under restrictive collimation protocols.

## Conclusion

In pediatric suspected foreign body ingestion, both collimation and exposure parameters substantially influence radiation dose. Abdominal radiographs demonstrated the highest radiation exposure compared with radiographs acquired using chest-based parameters, attributable predominantly to the abdominal exposure parameters employed, though the independent contribution of collimation cannot be fully disentangled given the study design. These findings suggest that a low-dose approach with reduced exposure settings for radiopaque, metallic foreign bodies is a useful strategy for dose reduction. Further dose savings may be achieved through a restrictive collimation strategy in selected clinical situations.

## Data Availability

Data is made available upon reasonable request.
